# Schwere Anaphylaxie auf Omalizumab

**DOI:** 10.1007/s00105-025-05542-9

**Published:** 2025-07-31

**Authors:** Franziska Keidel, Priscila Wölbing, Knut Schäkel

**Affiliations:** 1 Universitäts-Hautklinik Heidelberg, Ruprecht-Karls Universität Heidelberg, Im Neuenheimer Feld 440, 69120 Heidelberg, Deutschland; 2 Hautarztpraxis Wölbing, Darmstädter Str. 226–228, 64625 Bensheim, Deutschland

**Keywords:** Chronisch spontane Urtikaria, Omalizumab, Anaphylaxie, Polysorbat 20, Prick-Test, Chronic spontaneous urticaria, Omalizumab, Anaphylaxis, Polysorbate 20, Prick test

## Abstract

Ein Patient mit chronischer spontaner Urtikaria (CSU) erhielt eine Behandlung mit dem monoklonalen Antikörper Omalizumab, die eine erhebliche klinische Verbesserung bewirkte, jedoch wiederholt schwere anaphylaktische Reaktionen auslöste. Diese traten sowohl nach der Verabreichung des Medikaments als auch während eines Prick-Tests auf. Die genauen Mechanismen hinter den Reaktionen sind unklar, mögliche Ursachen könnten Zusatzstoffe wie Polysorbat 20 oder spezifische Antikörper sein. Der Fall verdeutlicht das seltene, aber ernste Risiko von Anaphylaxien bei Omalizumab und betont die Notwendigkeit einer engen Überwachung und Notfallbereitschaft.

## Abstract (en)

A patient with chronic spontaneous urticaria (CSU) received treatment with the monoclonal antibody omalizumab, which resulted in significant clinical improvement but repeatedly triggered severe anaphylactic reactions. These occurred both after administration of the medication and during a prick test. The exact mechanisms behind the reactions are unclear; possible causes could include additives such as polysorbate 20 or specific antibodies. The case highlights the rare but serious risk of anaphylaxis with omalizumab and emphasizes the need for close monitoring and emergency preparedness.

## Anamnese

Ein 39-jähriger kaukasischer Mann stellte sich in unserer Abteilung mit einer 6‑jährigen Vorgeschichte von chronischer spontaner Urtikaria (CSU) und einem allergischen Asthma vor. Der Patient berichtete von täglichem Juckreiz. Etwa 3‑mal/Monat kam es zu generalisierten Quaddeln, häufig begleitet von Angioödemen. Gelegentlich traten Symptome einer Anaphylaxie mit schwerer Dyspnoe auf, die eine wiederholte Einweisung in eine Notaufnahme erforderlich machten. Ein objektivierbarer Score konnte nicht erhoben werden.

Symptomatische Therapien mit Cetirizin 10 mg, Fexofenadin 180 mg oder Desloratadin 5 mg, jeweils bis zu 4‑mal täglich über mindestens 4 Wochen, waren unwirksam.

Aus der Anamnese des Patienten ging hervor, dass er vor Erstvorstellung mehrmals spontane anaphylaktoide Reaktionen ohne eruierbare Ursache hatte. Von einer Asthmaexazerbation berichtete der Patient nicht.

## Klinischer Befund

Bei Inspektion zeigte sich ein blandes Integument ohne den Nachweis von Quaddeln. Eine induzierbare Urtikaria wurde ausgeschlossen. Es gab keine Hinweise auf eine zugrunde liegende Infektionskrankheit oder einen Entzündungsherd. Die weitere Diagnostik bezüglich chronischer Urtikaria einschließlich der Untersuchung des Stuhls auf *Helicobacter-pylori*-Antigen ergab keine pathologischen Befunde. Außerdem gab es keinen Zusammenhang zwischen seinen Symptomen und der Exposition gegenüber bestimmten Medikamenten, Nahrungsmitteln oder physikalischen Reizen.

Bei Erstvorstellung zeigte sich ein erhöhtes Gesamt-IgE von 485 kU/l. Die Serumtryptase, Phadiatop Sx1 und ein Nahrungsmittelscreen Fx5 waren normwertig.

Der Patient berichtete von Unverträglichkeiten gegenüber Paprika und Nektarine. Hierbei kam es zu generalisiertem Juckreiz. Dies war weder reproduzierbar noch objektivierbar.

Die Vitalparameter waren normwertig. Die S_p_O_2_ lag bei 98 %.

## Diagnose

Chronische spontane Urtikaria mit begleitendem Angioödem.

## Therapie und Verlauf

Bei chronischer spontaner Urtikaria (CSU) begannen wir eine Behandlung mit dem monoklonalen Antikörper Omalizumab.

Eine Stunde nach der ersten subkutanen Injektion von 300 mg Omalizumab entwickelte der Patient einen generalisierten Juckreiz und Quaddeln, gefolgt von Heiserkeit, Dyspnoe und Husten mit einem Rückgang der Sauerstoffsättigung auf 90 %. Der Patient war zu diesem Zeitpunkt weiterhin zur Beobachtung in der Klinik. Bei weiterer Anamnese ergab sich kein möglicher Zusammenhang mit Nahrungsaufnahme, Medikamenten oder sonstiger Allergenexposition. Eine Überwachung auf der Intermediate-Care-Station war erforderlich. Der Patient erholte sich rasch nach Gabe von 0,5 mg Adrenalin, 1 mg Dimetinden und 250 mg Prednisolon. Er war im Hinblick auf die CSU in den folgenden 4 Wochen erstmals seit 6 Jahren symptomfrei.

Bei gutem Therapieerfolg bat der Patient um eine Fortsetzung der Omalizumab-Therapie. Wir beschlossen, eine reduzierte Dosis von 150 mg nach Prämedikation anzuwenden. Die Prämedikation bestand aus Prednisolon 25 mg 12 und 2 h vor der Anwendung des Medikaments und einer intravenösen Dimetinden-Dosis 30 min vor der Gabe von Omalizumab. Vor Gabe war der Patient beschwerdefrei.

Die anschließende Verabreichung von 150 mg Omalizumab wurde gut vertragen und war klinisch wirksam, da die CSU in den nächsten 2 Wochen unter Kontrolle war. Zwischen der ersten und zweiten Gabe lagen 10 Wochen (aufgrund von mehrmaliger Terminverschiebung durch den Patienten). Zwischen der zweiten und dritten Gabe lagen 4 Wochen.

Die dritte Verabreichung von 150 mg Omalizumab, wiederum unter Prämedikation, führte zu generalisiertem Juckreiz, Quaddeln und Dyspnoe. Eine symptomatische Behandlung mit intravenösen Kortikosteroiden und Antihistaminika führte zu einer raschen Linderung der Symptome.

Ein anschließend durchgeführter Prick-Test auf Omalizumab war negativ, doch 20 min nach der Anwendung entwickelte der Patient trockenen Husten und Übelkeit, gefolgt von einem Exanthem mit wenigen Quaddeln. Der Blutdruck sank auf 90/40 mm Hg, und die Sauerstoffsättigung fiel auf 87 %. Die Blutuntersuchung ergab zu diesem Zeitpunkt einen Serumtryptasewert von ≥200 µg/l. Innerhalb von 24 h sank die Serumtryptase auf 17 µg/l. Laborchemische und histologische Untersuchungen ergaben keinen Anhalt für eine systemische Mastozytose oder eine klonale Mastzellerkrankung. Die Tryptase wurde mehrfach kontrolliert und war, ausgenommen bei allergischen Reaktionen, immer normwertig. Unter intravenösem Prednisolon (250 mg), Dimetinden (1 mg) und Adrenalin (0,5 mg) besserte sich der Zustand rasch.

Ein Basophilenaktivierungstest mit Omalizumab war negativ. Der Test wurde gemäß etablierten Protokollen durchgeführt. Es wurde das Fertigpräparat Xolair® zur Testung verwendet. Eine anschließende Behandlung mit Ciclosporin A in einer Dosis von 2,5 mg pro kg Körpergewicht über 3 Monate konnte die CSU des Patienten nicht kontrollieren.

## Diskussion

Omalizumab, ein monoklonaler Anti-IgE-Antikörper, hat sich bei der Behandlung der chronischen spontanen Urtikaria (CSU) als wirksam erwiesen [1, 7]. Auch bei der Vorbeugung von Anaphylaxie bei Patienten mit chronischer spontaner Urtikaria zeigte sich ein Nutzen [10]. Allerdings kann seine Anwendung selbst mit schweren Nebenwirkungen, einschließlich Anaphylaxie, verbunden sein.

Eine durch die Verabreichung von Omalizumab ausgelöste Anaphylaxie ist selten und tritt bei etwa 0,2 % der behandelten Patienten auf [4]. Die Daten beziehen sich hier auf Studien mit Asthmapatienten. Am häufigsten wurde über Anaphylaxie bei der Behandlung von Asthma bronchiale berichtet, aber auch nach der Verabreichung von Omalizumab zur Behandlung von CSU [3, 4, 6, 9]. Zu den prädisponierenden Faktoren für die Entwicklung einer Anaphylaxie nach einer Omalizumab-Behandlung gehören Asthma bronchiale, Nahrungsmittelallergien oder eine Anaphylaxie in der Vorgeschichte – alle diese Faktoren waren bei unserem Patienten vorhanden [5].

Die Ursache der Omalizumab-assoziierten Anaphylaxie ist noch unklar. Es wird jedoch vermutet, dass möglicherweise Polysorbat, ein Zusatzstoff, der zur Verbesserung der Löslichkeit des Medikaments in der Omalizumab-Formulierung verwendet wird, zu Überempfindlichkeitsreaktionen führen kann [8, 11]. Andere vermuten, dass bereits vorhandene oder entwickelte antiallotypische oder antiidiotypische Antikörper (IgE oder IgG) gegen Omalizumab die Überempfindlichkeitsreaktion verursachen [2].

Unseres Wissens ist dies der erste Fall einer Anaphylaxie nach einer Exposition gegenüber Omalizumab nicht nur in einer Behandlungssituation, sondern auch während eines klassischen Prick-Tests. Sein basaler Tryptasewert lag innerhalb des Referenzbereichs, stieg jedoch nach dem anaphylaktischen Ereignis sprunghaft auf Werte von über 200 µg/l an, was darauf hindeutet, dass eine Degranulation von Basophilen und Mastzellen stattgefunden hat.

Zusammenfassend verdeutlicht dieser Fall das Risiko einer schweren Anaphylaxie durch Omalizumab. Überwachung und Vorbereitung auf eine Notfallbehandlung sind unerlässlich.

## Fazit für die Praxis

- Omalizumab ist ein monoklonaler Antikörper, der für die Behandlung der therapierefraktären chronisch spontanen Urtikaria eine effektive Therapie darstellen kann.
- Bei etwa 0,2 % der Therapien kann eine allergische Reaktion bis hin zu einer schweren Anaphylaxie auftreten. Die Daten beziehen sich hier auf Studien mit Asthmapatienten. Die ersten Gaben sollten unter Überwachung und Notfallbereitschaft durchgeführt werden.
